# Supplementary material for: Transient acidosis while retrieving a fear-related memory enhances its lability
Source: eLife. 2017 Jun 26;6:e22564. doi: 10.7554/eLife.22564 (PMC5484615; doi:10.7554/eLife.22564)
Supplement: Figure 1—source data 1. — Chart indicates the conditions used for contexts X, Y, Z, and ZZ. DOI: http://dx.doi.org/10.7554/eLife.22564.003 [file elife-22564-fig1-data1.docx]

|  | **Odor** | **Light** | **Floor** | **Roof/Wall** |
| --- | --- | --- | --- | --- |
| **Context X** | Bleach (1%) | + | Metal bars | Square |
| **Context Y** | Peppermint (0.25%) | - | White smooth floor | Black triangle roof |
| **Context Z** | Anise (0.25%) | - | Metal bars | Round wall |
| **Context ZZ** | Vanilla (0.25%) | - | Metal grids | Round roof |

**Figure 1- source data 1**

**Contexts for experiments.** Chart indicates the conditions used for contexts X, Y, Z, and ZZ.
